# Supplementary material for: Cross-Sectional Association between Hypercholesterolemia and Knee Pain in the Elderly with Radiographic Knee Osteoarthritis: Data from the Korean National Health and Nutritional Examination Survey
Source: J Clin Med. 2021 Mar 1;10(5):933. doi: 10.3390/jcm10050933 (PMC7957475; doi:10.3390/jcm10050933)
Supplement: Supplementary file 1 [file jcm-10-00933-s001.pdf]

Supplementary Materials

Table S1. Result of multivariable ordinal logistic regression analysis for knee pain (numeric rating scale) in subjects with radiographic OA.

Variables	Univariable			Multivariable		
	OR	95% CI	*p*-value	OR	95% CI	*p*-value
Female sex	2.79	2.37–3.29	**<0.001**	1.50	1.18–1.91	**0.001**
Age	1.04	1.02–1.05	**<0.001**	1.01	0.99–1.02	0.444
BMI	1.07	1.04–1.09	**<0.001**	1.04	1.01–1.07	**0.011**
Residential area (rural)	1.25	1.05–1.48	**0.010**	1.07	0.89–1.29	0.497
Household income			**<0.001**			**0.020**
Quartile 4 (high)	1			1		
Quartile 3	1.06	0.83–1.36		1.09	0.84–1.42	
Quartile 2	1.45	1.15–1.84		1.35	1.05–1.74	
Quartile 1 (low)	1.69	1.37–2.09		1.38	1.08–1.76	
Alcohol consumption			**<0.001**			0.638
High	1			1		
Moderate	1.29	0.91–1.83		1.00	0.64–1.54	
Low	2.20	1.66–2.92		1.21	0.78–1.62	
Physical activity			**<0.001**			0.796
High	1			1		
Moderate	1.16	0.96–1.41		1.09	0.86–1.38	
Low	1.46	1.22–1.74		1.05	0.85–1.30	
Education attainment			**<0.001**			**<0.001**
Graduate school	1			1		
University	1.26	0.37–4.34		1.72	0.45–6.60	
College	1.47	0.26–8.34		1.32	0.21–8.22	
High school	2.38	0.81–7.03		2.13	0.65–6.96	
Middle school	4.85	1.66–14.19		4.01	1.24–12.96	
Elementary school	6.91	2.42–19.92		4.60	1.45–14.61	
Less than elementary school	11.71	4.02–34.06		6.88	2.10–22.55	
None	8.85	2.71–28.91		4.02	1.09–14.86	
KL grade			**<0.001**			**<0.001**
Grade 4	1			1		
Grade 3	0.35	0.29–0.42		0.37	0.30–0.45	
Grade 2	0.15	0.12–0.18		0.21	0.17–0.26	
Metabolic syndrome	1.28	1.09–1.50	**0.002**	1.03	0.85–1.25	0.752
Hypercholesterolemia	1.37	1.13–1.65	**0.001**	1.24	1.02–1.52	**0.033**

OA, osteoarthritis; OR, odds ratio; CI, confidence interval; BMI, body mass index; KL, Kellgren–Lawrence; Bold, *p*–value < 0.05.

Table S2. Result of multivariable ordinal logistic regression analysis for knee pain (numeric rating scale) in subjects without radiographic OA.

Variables	Univariable			Multivariable		
	**OR**	**95% CI**	***p*-value**	**OR**	**95% CI**	***p*-value**
Female sex	3.07	2.47–3.82	**<0.001**	2.05	1.49–2.82	**<0.001**
Age	1.03	1.01–1.05	**0.003**			0.802
BMI	1.07	1.03–1.11	**0.002**			**0.003**
Residential area (rural)	1.44	1.15–1.80	**0.002**	1.31	1.02–1.67	**0.032**
Household income			**<0.001**			0.172
Quartile 4 (high)	1			1		
Quartile 3	1.42	0.96–2.10		1.25	0.82–1.89	
Quartile 2	1.77	1.22–2.56				
Quartile 1 (low)	2.19	1.55–3.10				
Alcohol consumption			**<0.001**			0.712
High	1			1		
Moderate	1.41	0.89–2.23		1.16	0.71–1.88	
Low	2.35	1.64–3.36		1.19	0.79–1.81	
Current smoking	0.52	0.38–0.71	**<0.001**	0.80	0.55–1.16	0.235
Physical activity			**0.049**			0.819
High	1			1		
Moderate	1.24	0.92–1.67		1.10	0.81–1.51	
Low	1.40	1.07–1.84		1.08	0.80–1.46	
Education attainment			**<0.001**			**<0.001**
Graduate school	1			1		
University	0.35	0.12–1.02		0.33	0.11–1.04	
College	0.50	0.14–1.78		0.33	0.08–1.31	
High school	0.56	0.23–1.38		0.45	0.17–1.16	
Middle school	1.18	0.47–2.91		0.82	0.30–2.19	
Elementary school	1.69	0.70–4.08		0.89	0.34–2.32	
Less than elementary school	3.62	1.46–8.96		1.74	0.64–4.71	
None	2.44	0.69–8.69		1.55	0.40–5.97	
Hypercholesterolemia	1.33	1.03–1.72	**0.027**	1.14	0.88–1.49	0.329

OA, osteoarthritis; OR, odds ratio; CI, confidence interval; BMI, body mass index; Bold, *p*-value < 0.05.

Table S3. Result of multivariable ordinal logistic regression analysis for severity of radiographic OA (KL grade).

Variables	Univariable			Multivariable		
	OR	95% CI	*p*-value	OR	95% CI	*p*-value
Female sex	2.86	2.62–3.12	**<0.001**	2.41	2.13–2.73	**<0.001**
Age	1.08	1.07–1.09	**<0.001**	1.09	1.08–1.11	**<0.001**
BMI	1.11	1.10–1.13	**<0.001**	1.15	1.13–1.17	**<0.001**
Residential area (rural)	1.42	1.25–1.62	**<0.001**	1.32	1.15–1.52	**<0.001**
Household income			**<0.001**			0.814
Quartile 4 (high)	1			1		
Quartile 3	1.06	0.91–1.24		1.03	0.88–1.20	
Quartile 2	1.23	1.05–1.43		1.08	0.92–1.27	
Quartile 1 (low)	1.55	1.34–1.80		1.03	0.87–1.21	
Alcohol consumption			**<0.001**			0.606
High	1			1		
Moderate	1.14	0.94–1.38		1.04	0.86–1.25	
Low	1.94	1.65–2.27		1.09	0.91–1.30	
Current smoking	0.56	0.49–0.64	**<0.001**	0.97	0.83–1.13	0.682
Physical activity			**<0.001**			
High	1			1		
Moderate	1.12	0.99–1.27		1.09	0.95–1.25	
Low	1.33	1.18–1.49		1.10	0.97–1.25	
Education attainment			**<0.001**			**<0.001**
Graduate school	1			1		
University	1.00	0.70–1.42		0.96	0.64–1.42	
College	1.01	0.59–1.72		1.17	0.66–2.07	
High school	1.50	1.07–2.09		1.35	0.93–1.98	
Middle school	1.83	1.29–2.58		1.43	0.96–2.13	
Elementary school	2.85	2.03–3.98		1.67	1.13–2.48	
Less than elementary school	4.92	3.49–6.93		1.74	1.16–2.62	
None	5.80	3.29–10.23		1.84	0.94–3.60	
Hypertension	1.53	1.39–1.69	**<0.001**	1.16	1.03–1.31	**0.012**
Metabolic syndrome	1.43	1.28–1.58	**<0.001**	1.15	1.02–1.29	**0.018**
Hypercholesterolemia	1.13	1.00–1.29	0.056			

OA, osteoarthritis; OR, odds ratio; CI, confidence interval; BMI, body mass index; KL, Kellgren-Lawrence; Bold, *p*-value < 0.05.
